# Sunitinib significantly suppresses the proliferation, migration, apoptosis resistance, tumor angiogenesis and growth of triple-negative breast cancers but increases breast cancer stem cells

**DOI:** 10.1186/2045-824X-6-12

**Published:** 2014-06-01

**Authors:** Edmund Chinchar, Kristina L Makey, John Gibson, Fang Chen, Shelby A Cole, Gail C Megason, Srinivassan Vijayakumar, Lucio Miele, Jian-Wei Gu

**Affiliations:** 1 Cancer Institute, University of Mississippi Medical Center, 2500 North State Street, 39216-4505 Jackson, MS, USA; 2 Department of Physiology & Biophysics, University of Mississippi Medical Center, Jackson, MS 39216, USA; 3 Children’s Cancer Center, University of Mississippi Medical Center, Jackson, MS 39216, USA

**Keywords:** Sunitinib, Basal-like triple-negative breast cancer, Xenografts, Angiogenesis, Proliferation, Migration, Apoptosis, Breast cancer stem cell, Notch-1

## Abstract

The majority of triple-negative breast cancers (TNBCs) are basal-like breast cancers. However there is no reported study on anti-tumor effects of sunitinib in xenografts of basal-like TNBC (MDA-MB-468) cells. In the present study, MDA-MB-231, MDA-MB-468, MCF-7 cells were cultured using RPMI 1640 media with 10% FBS. Vascular endothelia growth factor (VEGF) protein levels were detected using ELISA (R & D Systams). MDA-MB-468 cells were exposed to sunitinib for 18 hours for measuring proliferation (3H-thymidine incorporation), migration (BD Invasion Chamber), and apoptosis (ApopTag and ApoScreen Anuexin V Kit). The effect of sunitinib on Notch-1 expression was determined by Western blot in cultured MDA-MB-468 cells. 10^6^ MDA-MB-468 cells were inoculated into the left fourth mammary gland fat pad in athymic nude-foxn1 mice. When the tumor volume reached 100 mm^3^, sunitinib was given by gavage at 80 mg/kg/2 days for 4 weeks. Tumor angiogenesis was determined by CD31 immunohistochemistry. Breast cancer stem cells (CSCs) isolated from the tumors were determined by flow cytometry analysis using CD44^+^/CD24^-^ or low. ELISA indicated that VEGF was much more highly expressed in MDA-MB-468 cells than MDA-MB-231 and MCF-7 cells. Sunitinib significantly inhibited the proliferation, invasion, and apoptosis resistance in cultured basal like breast cancer cells. Sunitinib significantly increased the expression of Notch-1 protein in cultured MDA-MB-468 or MDA-MB-231 cells. The xenograft models showed that oral sunitinib significantly reduced the tumor volume of TNBCs in association with the inhibition of tumor angiogeneisis, but increased breast CSCs. These findings support the hypothesis that the possibility should be considered of sunitinib increasing breast CSCs though it inhibits TNBC tumor angiogenesis and growth/progression, and that effects of sunitinib on Notch expression and hypoxia may increase breast cancer stem cells. This work provides the groundwork for an innovative therapeutic strategy in TNBC therapy by using sunitinib plus γ-secretase inhibitor to simultaneously target angiogenesis and CSC.

## Introduction

Triple-negative breast cancer (TNBC) refers to any breast cancer that does not express the genes for estrogen receptor (ER), progesterone receptor (PR) and Her2/neu [1]. TNBC accounts for >15% of breast cancer [2], and 39% in African American premenopausal women with breast cancer [3]. TNBCs exhibit a high level of molecular heterogeneity, and are biologically aggressive: a poor prognostic factor for disease-free and overall survival in the adjuvant and neoadjuvant setting, a more aggressive clinical course in the metastatic setting, and no effective specific targeted therapy [1,2]. TNBCs comprise the basal and claudin-low molecular subtypes. The majority of TNBCs (approximately 80%) are basal-like breast cancers [4]. The signal-transduction pathways involving vascular endothelial growth factor receptor (VEGFR), platelet-derived growth factor receptor (PDGFR), stem-cell factor receptor (KIT), and colony stimulating factor-1 receptor (CSF-1R) have been implicated in breast cancer pathogenesis [5-10]. VEGFR and KIT have shown to be associated with TNBCs [10-13]. Sunitinib is an inhibitor of receptor tyrosine kinases that include VEGFR, PDGFR, KIT, and CSF1R [6,11,14]. We previously reported that sunitinib targeted the paracrine and autocrine effects of VEGF on breast cancer to suppress tumor angiogenesis, proliferation and migration in a mouse ER-positive breast cancer model [11]. There were several reports that sunitinib inhibited tumor angiogenesis and tumor growth in xenografts of the claudin-low TNBC (MDA-MB-231) cells [15-17]. In a phase II study in patients with heavily pretreated metastatic breast cancer, 15% of patients (three of 20) with TNBC achieved partial responses following treatment with single-agent sunitinib [18]. However, there is no reported study on anti-tumor effects of sunitinib in xenografts of the basal-like TNBC (MDA-MB-468) cells.

Sunitinib has been used as anticancer treatments in several tumor types including breast cancer [19], however clinical observations indicate this therapy may have limited efficacy. When anti-angiogenic agents are administered on an intermittent schedule, such as with sunitinib (4 wk on, 2 wk off), tumor regrowth is sometimes seen during drug-free periods [18] or upon discontinuation of the treatment [20]. Although anti-angiogenic agents produce inhibition of primary tumor growth, lasting responses are rare, with only a moderate increases in progression-free survival and little benefit in overall survival [21]. Anti-angiogenic agents generate intratumoral hypoxia modulating the metastatic process [22] and stimulating cancer stem cells (CSC) [23,24]. Cancer stem cells (CSCs) are cells that have the ability to self-renew and give rise to differentiated tumor cells, and constitute a rare subpopulation in a tumor mass. CSCs are thought to play a role in recurrence and metastasis of TNBC [25]. Various experiments support that the Notch pathway is critical in controlling the fate of CSC in breast cancer [25,26] and that anti-angiogenic therapy may actually activate Notch and preserve CSC [27]. It is therefore possible that sunitinib may induce breast cancer CSC and activate the Notch pathway.

We hypothesize that sunitinib can suppress basal-like TNBC tumor angiogenesis and growth/progression via inhibition of paracrine and autocrine effects of VEGF, and that sunitinib-induced tumor hypoxia may increase breast cancer stem cells. Thus, the present study aimed to determine the following: 1) whether VEGF is highly expressed in MDA-MB-468 cells, compared to MCF-7 and MDA-MB-231 cells; 2) whether sunitinib inhibits the proliferation, migration, apoptosis resistance of cultured MDA-MB-468 cells; 3) whether oral sunitinib treatment suppresses tumor angiogenesis and growth in the basal-like TNBC (MDA-MB-468) xenografts; 4) whether sunitinib increases the percentage of breast cancer stem cells in the xenografts; and 5) whether sunitinib increases the expression of Notch-1 in MDA-MB-468 cells. The effects of sunitinib on claudin-low TNBC MDA-MB-231 xenografts and cell cultures were also tested.

## Materials and methods

### Chemicals and cell lines

Sunitinib was purchased from LC Laboratories (Woburn, MA). Human estrogen-receptor positive breast cancer (MCF-7) cells, human claudin-low triple-negative breast cancer (MDA-MB-231) cells, and basal-like breast cancer (MDA-MB-468) cells were purchased from the American Type Culture Collection (Rockville, MD). All breast cancer cells were maintained as monolayer cultures in RPMI Medium 1640 (GIBCO) supplemented with 10% FBS (HyClone), 100 U/ml penicillin, 100 μg/ml streptomycin, and 0.25 μg/ml amphotericin B, and incubated at 37°C in a humidified 5% CO_2_/air injected atmosphere. Sunitinib was suspended in vehicle containing carboxymethylcellulose sodium (United States Pharmacopia; 0.5% wt/vol, NaCl 1.8% wt/vol); Tween 80 0.4% wt/vol), benzyl alcohol 0.9% wt/vol), and deionized water adjusted to pH 6.0. Sunibinib was prepared weekly and kept at 4°C.

### Animal protocols

The protocols were carried out according to the guidelines for the care and use of laboratory animals implemented by the National Institutes of Health and the Guidelines of the Animal Welfare Act and were approved by the University of Mississippi Medical Center’s Institutional Animal Care and Use Committee. Eight female athymic nude-Foxn1 mice at 10 weeks of age were purchased from Harlan Laboratories (Indianapolis, IN). The mice were allowed to acclimate for 2 weeks with standard chow diet (Teklad, Harlan Sprague Dawley; Indianapolis, IN) and tap water before beginning the experiments. The twelve week old female mice (n = 8) were inoculated with 10^6 MDA-MB-468 cells suspended in 100 μl of phosphate-buffered saline with matrigel (BD Bioscience, Bedford, MA) into the left fourth mammary gland fat pad. Two weeks after the inoculation, the tumor volume reached around 100 mm^3^. Then 4 mice received sunitinib given by gavage at 80 mg/kg/2 days for 4 weeks and other 4 mice received the vehicle only as the control group. For MDA-MB-231 xenografts, the twelve week old female mice (n = 8) were inoculated with 10^6 MDA-MB-231 cells into the left fourth mammary gland fat pad. When the tumor volume reached around 500 mm3, four female athymic nude-Foxn1 mice received sunitinib given by gavage at 80 mg/kg/2 days for 4 weeks and the other 4 mice received the vehicle only as the control group. The body weight of the mice was monitored weekly. Tumor size was monitored every other day in two perpendicular dimensions parallel with the surface of the mice using dial calipers. At the end of the experiment, blood samples and tumors were collected to measure VEGF expression using ELISA and average microvascular density (AMVD) or capillary density (CD) using CD31 immunohistochemistry.

### Morphometric analysis of angiogenesis in tumors

The quantification of blood vessels in the tumors of xenografts with MDA-MB-468 cells or MDA-MB-231 cells was determined with the modification of a previously reported method [11,28]. Briefly, the tissues were fixed in 4% neutrally buffered paraformaldehyde. Consecutive thin cryosections (5 μm) of OCT compound (Sakura Finetek, Torrance, CA) embedded tissue samples were fixed in acetone at 4°C for 10 min. After washing in phosphate buffered saline (PBS), the sections were first treated with 3% H_2_O_2_ for 10 minutes to block endogenous peroxidase activity and then were blocked with normal rabbit serum. Next, the sections were washed in PBS and incubated with rat anti-mouse CD31 (PECAM-1) monoclonal antibody (BD Pharmingen, San Diego, CA) at a 1:200 dilution overnight at 4°C. Negative controls were incubated with the rat serum IgG at the same dilution. All sections were washed in PBS containing 0.05% Tween-20, and were then incubated with a 2nd antibody, mouse anti-rat IgG (Vector laboratories, Burlingame, CA) at a 1:200 dilution for 1 hour at room temperature, again followed by washing with PBS containing 0.05% Tween-20. The sections were incubated in a 1:400 dilution of Extravadin Peroxidase (Sigma, St. Louis, MO) for 30 min. After washing in PBS containing 0.05% Tween-20, the sections were incubated in peroxidase substrate (Vector laboratories, Burlingame, CA) for 5 min. The sections were washed in PBS containing 0.05% Tween-20 and were counterstained with hematoxylin. A positive reaction was indicated by a brown staining. The microvascular vessels or capillary density (CD) were quantified by manual counting under light microscopy. A microscopic field (0.7884 mm2) was defined by a grid laced in the eye-piece. At least 20 microscopic fields were randomly acquired from each tumor for analysis. Any endothelial cell or cell cluster showing antibody staining and clearly separated from an adjacent cluster was considered to be a single, countable microvessel or capillary. The value of the average microvascular density (AMVD) or capillary density (CD) was determined by calculating the mean of the vascular counts per mm^2^ obtained in the microscopic fields for each tissue sample.

### Flow cytometry

The tumor cells in a single cell suspension were isolated from the each xenograft within 2 hours by using the gentleMACs Dissociator and Tumor Dissociation Kit (Miltenyi Biotec Inc., Auburn, CA) according to the manufacturer’s guidelines. 0.5 × 10^6^ cells per sample for flow cytometry analysis were as follows: a) unstained; b) stained with mouse IgG1-PE/-FITC; c) stained with anti-human CD44-PE; d) stained with anti-human CD24-FITC; and e) stained with anti-human CD44-PE/CD24-FITC (Miltenyi Biotec Inc., Auburn, CA). The fluorescence intensity of these cell samples was analyzed by the Gallios flow cytometer (Beckman Coulter, Inc., Brea, CA). The ALDEFLUOR kit (Stemcell Technologies) was used for the identification of cancer stem cells from MDA-MB-231 xenografts by flow cytometry analysis.

### Measurements of protein levels of VEGF by ELISA

Protein levels of VEGF in cultured MCF-7, MDA-MB-231, and MDA-MB-468 cells were determined using mouse VEGF ELISA kits (R & D Systems, Minneapolis, MN), according to the manufacturer’s instructions. The total proteins of cultured MCF-7, MDA-MB-231, or MDA-MB-468 cells were extracted using NE-PER Cytoplasmic Extraction Reagents (Pierce, Rockford, IL), according to the manufacturer’s protocol. Protein levels of VEGF in these cells were determined in the cultured media 18 hrs after the incubation. The total protein concentration of cultured MCF-7, MDA-MB-231 or MDA-MB-468 cells was determined using a Bio-Rad Protein Assay (Bio-Rad Laboratories, Hercules, CA). The protein concentrations of VEGF were normalized and expressed as pictograms per milligram of total cellular protein.

### Proliferation assay

The MDA-MB-468 or MDA-MB-231 cells were seeded into 6-well tissue culture plates using RPMI Medium 1640 (GIBCO) supplemented with 10% FBS (HyClone), 100 U/ml penicillin, 100 μg/ml streptomycin, and 0.25 μg/ml amphotericin B, and incubated at 37°C in a humidified 5% CO_2_/air injected atmosphere. When the monolayer reached about 80% confluence, the cells were washed with PBS and incubated with fresh RPMI Medium 1640 with 10% FBS in the absence or presence of sunitinib (0, 1, 5, or 10 μmol/L) for 18 hours. 3H-thymidine incorporation assay was used to determine the cell proliferation during the last 6 hours of incubation as previously described [29].

### Migration assay

Migration was determined using BD BioCoat Matrigel Invasion Chamber (BD Bioscience Discovery Labware, Sedford, MA) according to a previous study, in which only invasive cells digested the matrix and moved through the insert membrane [29]. 1 × 10^5^ MDA-MB-468 cells per well in 0.5 ml RPMI Medium 1640 were seeded in the matrigel-coated upper compartment (insert) of a Transwell (24-well format, 8-μm pore) in the absence or presence of sunitinib (1 μmol/L). Medium with 10% FBS was added to the lower part of the well. After overnight incubation at 37°C and 5% CO_2_, cells on the upper surface of the insert were removed using a cotton wool swab. Migrated cells on the lower surface of the insert were stained using DiffQuit (Dada Behring, Düdinen, Switzerland). Images of migrated cells were taken and the number of migrated cells was counted using a microscope (Leica, Germany) in a 20× objective.

### Apoptosis assay

TUNEL staining was performed using the ApopTag Peroxidase *In Situ* Apoptosis Detection Kit (Millipore) according to the manufacturer’s instructions. Cultured MDA-MB-468 cells were treated with Sunitinib (5 μmol/L) or the vehicle only as the control group for 24 hours. The cells were fixed in 10% neutral buffered formalin and stained for DNA strand breaks associated with apoptosis following the manufacturer’s instructions. The cells were counterstained with methyl green (Vector Laboratories). The ApoScreen Anuexin V Apoptosis Kit (Beckman Coulter) was also used to detect apoptotic cells. Cultured MDA-MB-468 cells were treated with Sunitinib (5 μmol/L) or the vehicle only as the control group for 24 hours. The cells in single cell suspensions were collected, stained with Anuexin V-FITC, and analyzed by flow cytometry according to the manufacturer’s guidelines.

### Western blot

Cultured MDA-MB-468 or MDA-MB-231 cells were treated with Sunitinib (0.1 and 1 μmol/L) or the vehicle for 24, 48, and 72 hours. Western blot analysis was performed as previously described [30]. Briefly, Whole cell extracts were prepared by lysing cells in RIPA buffer containing a mixture of protease and phosphatase inhibitor cocktails (Thermo Scientific, Rockford, IL) followed by sonication and centrifugation. Protein concentration was determined using a Bradford Assay (BioRad Laboratories, Hercules, CA). 50 μg of protein was loaded onto a 4-20% gradient (SDS/PAGE) precast gel (BioRad Laboratories, Hercules, CA), transferred to a nitrocellulose membranes, and blocked with Odyssey blocking buffer (LI-COR Biosciences, Lincoln, NE). The membranes was then incubated anti-Notch-1 (C-20) antibody (Santa Cruz Biotechnology) (1:500) dilution in 10 ml of blocking buffer containing 0.15% Tween 20, and washed with PBST buffer containing 0.1% Tween 20. IRdye 680 secondary antibodies were used to visualize and quantify the protein bands by an Odyssey scanner (LI-COR Biosciences, Lincoln, NE). β-actin expression (Sigma-Aldrich) was evaluated as a control for protein loading. Differences in protein expression were determined by densitometry analysis using ImageJ Software (National Institutes of Health, USA).

### Statistical analysis

All determinations were performed in duplicated sets. Where indicated, data is presented as mean ± SE. Statistically significant differences in mean values between the two groups were tested by an unpaired Student’s t-test. Linear regression was performed by the correlation analysis between two continuous variables. A value of P < 0.05 was considered statistically significant. All statistical calculations were performed using SPSS software (SPSS Inc., Chicago, IL).

## Results

### Oral administration of sunitinib suppresses the progression of TNBC tumor growth in nude mice

**Figure 1:**
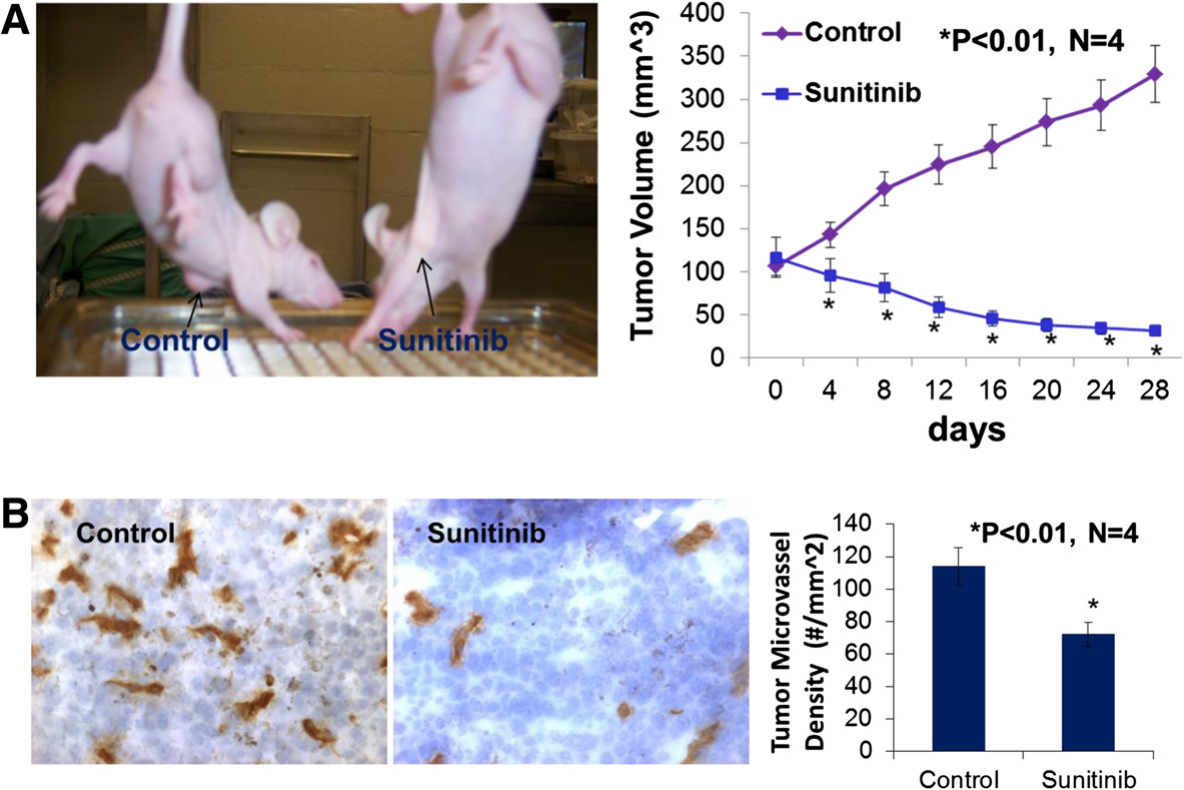
**Sunitinib treatment significantly inhibited tumor growth and tumor angiogenesis of the basal-like triple-negative breast cancer.** Oral sunitinib significantly suppressed the basal-like TNBC growth curve of tumor volume in MDA-MB-468/xenografts **(A)**. When the tumor volume reached around 100 mm^3^, four female athymic nude-Foxn1 mice received sunitinib given by gavage at 80 mg/kg/2 days for 4 weeks and the other 4 mice received the vehicle only as the control group. At the conclusion of the experiment, the tumor volume was significantly reduced by 90.4% (p < 0.01; n = 4) in the sunitinib-treated group in contrast to the control group, which was consistent with the reduction in tumor weight in the sunitinib-treated group compared to the control group (31 ± 0.6 vs. 294 ± 28 mg; P <0.01). The digital images of CD31 staining of the basal-like TNBC tumors showed that the sunitinib-treated tumor had fewer microvessels than the control tumor **(B)**. Morphometric analysis **(B)** indicated that sunitinib- treatment caused a significant decrease in average microvessel density (the number of microvessels per mm^2^ area) of the basal-like TNBC tumors when compared to the control tumors (72 ± 8 vs. 114 ± 10 microvessels number per mm^2^; n = 4; p < 0.01).

**Figure 2:**
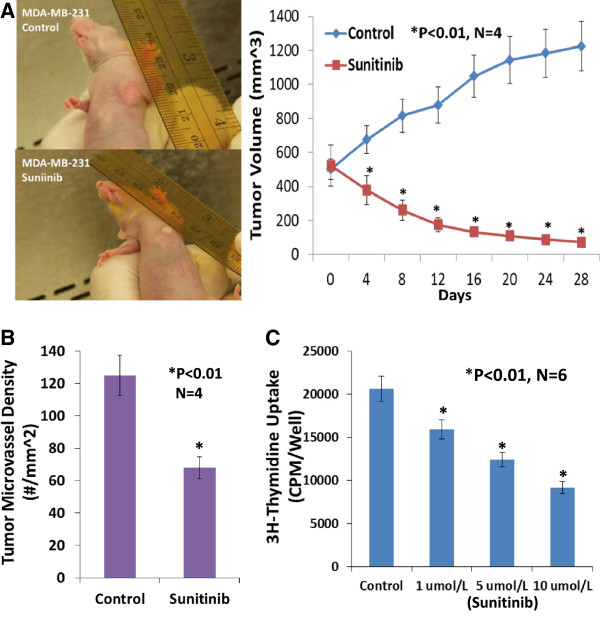
**Sunitinib treatment significantly inhibited tumor growth, tumor angiogenesis, and the proliferation of the claudin-low triple negative breast cancer.** Oral sunitinib at 80 mg/kg/2 days for 4 weeks significantly suppressed the claudin-low TNBC growth curve of tumor volume **(A)** and tumor angiogenesis **(B)** in MDA-MB-231/xenografts. When the tumor volume reached around 500 mm3, four female athymic nude-Foxn1 mice received sunitinib given by gavage at 80 mg/kg/2 days for 4 weeks and the other 4 mice received the vehicle only as the control group. In the end, the tumor volume was significantly reduced by 94% (P < 0.01; n = 4) in the sunitinib-treated group in contrast to the control group, which was consistent with the inhibition of tumor angiogenesis **(B)**. Sunitinib- treatment caused a significant decrease in average microvessel density (the number of microvessels per mm^2^ area) of the claudin-low TNBC tumors when compared to the control tumors (68 ± 9 vs. 125 ± 16 microvessels number per mm^2^; n = 4; p < 0.01). 3H-thymidine incorporation assay indicated that sunitinib-treatment caused a dose-related inhibition on proliferation in cultured MDA-MB-231 cells, by 23% at 1 μmol/L, by 40% at 5 μmol/L, and 55% at 10 μmol/L, compared to the control group (n = 6; P < 0.01), respectively **(C)**.

When the tumor volume reached around 100 mm^3^ in the basal-like TNBC (MDA-MB-468) xenografts, four female athymic nude-Foxn1 mice received sunitinib given by gavage at 80 mg/kg/2 days for 4 weeks and the other 4 mice received the vehicle only as the control group. On the day 4 of treatment, the tumor volume was significantly reduced by 32.9% (p < 0.01) in the sunitinib-treated group in contrast to the control group (Figure 1A). At the conclusion of the experiment, the tumor volume was significantly reduced by 90.4% (p < 0.01) in the sunitinib-treated group in contrast to the control group (Figure 1A), which was consistent with the reduction in tumor weight in the sunitinib-treated group compared to the control group (31 ± 0.6 vs. 294 ± 28 mg; P <0.01). For MDA-MB-231 xenografts, when the tumor volume reached around 500 mm^3^, four female athymic nude-Foxn1 mice received sunitinib given by gavage at 80 mg/kg/2 days for 4 weeks and the other 4 mice received the vehicle only as the control group. In the end, the tumor volume was significantly reduced by 94% (p < 0.01; n = 4) in the sunitinib-treated group in contrast to the control group (Figure 2). Clearly, oral sunitinib at 80 mg/kg/2 days for 4 weeks very significantly inhibited tumor growth in the basal-like TNBC (MDA-MB-468) or the claudin-low TNBC (MDA-MB-231) xenografts.

### Sunitinib-treatment inhibits tumor angiogenesis of the basal-like or clauding-low TNBC in mice

Growth and expansion of tumor mass is mainly dependent on angiogenesis because neovascularization contributes rapid tumor growth by providing an exchange of nutrients, oxygen and paracrine stimulus of the tumor. Therefore, in this study, we used a morphometric analysis of immunohistochemical staining for CD31 to determine the effect of sunitinib on tumor angiogenesis of the basal-like TNBC. Representative images of CD31 staining of the breast cancer tumors showed that the sunitinib-treated tumor had fewer microvessels than the control tumor (Figure 1B). Morphometric analysis (Figure 1B) indicated that sunitinib treatment caused a significant decrease in average microvessel density (the number of microvessels per mm^2^ area) of the basal-like TNBC tumors when compared to the control tumors (72 ± 8 vs. 114 ± 10 microvessels number per mm^2^; n = 4; p < 0.01). For MDA-MB-231 xenografts (Figure 2), sunitinib- treatment caused a significant decrease in average microvessel density (the number of microvessels per mm2 area) of the claudin-low TNBC tumors when compared to the control tumors (68 ± 9 vs. 125 ± 16 microvessels number per mm^2^; n = 4; p < 0.01). These results suggest that the pronounced decrease in tumor angiogenesis is associated with the decrease in tumor size found in the sunitinib treated groups compared to those in the control groups.

### VEGF expression is higher in the basal-like TNBC (MDA-MB-468) than MDA-MB-231and MCF-7 cells

**Figure 3:**
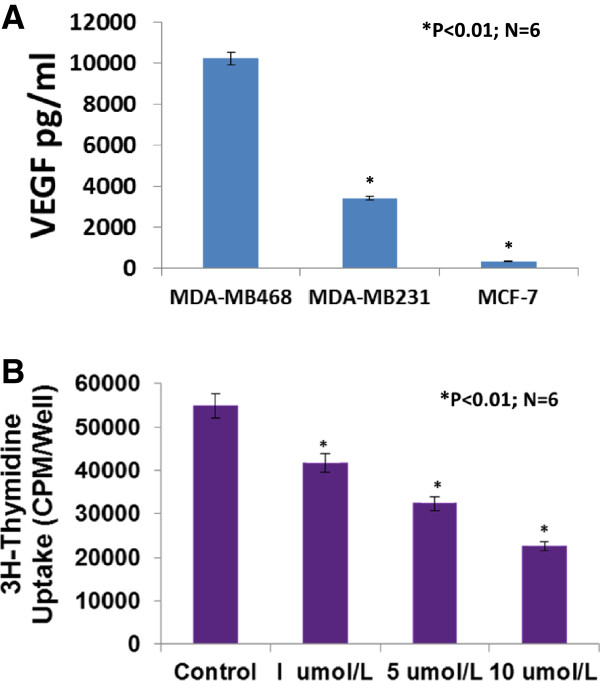
**VEGF protein was highly expressed in cultured MDA-MB-468 cells in which sunitinib-treatment caused a dose-related inhibition on the proliferation.** Figure **A** showed that VEGF protein was more expressed in MDA-MB-468 cells than MDA-MB-231 cells (>3 fold, P < 0.01, n = 6; 10257 ± 212 vs. 3408 ± 136 pg/mg) or MCF-7 cells (>30 fold, P < 0.01, n = 6; 10257 ± 212 vs. 336 ± 15 pg/mg). 3H-thymidine incorporation assay indicated that sunitinib-treatment caused a dose-related inhibition on proliferation in cultured MDA-MB-468 cells, by 24% at 1 μmol/L, by 41% at 5 μmol/L, and 59% at 10 μmol/L, compared to the control group (n = 6; P < 0.01), respectively **(B)**.

VEGF is involved in promoting breast cancer progression [11,31]. VEGF and its receptors are expressed in MCF-7 and MDA-MB-231 cells [11,32], however, it has not been reported whether VEGF is expressed differentially in MDA-MB-468, MDA-MB-231 and MCF-7 cells. We examined the expression of VEGF protein in cultured MDA-MB-468, MDA-MB-231 and MCF-7 cells using ELISA assay. Figure 3A shows that VEGF protein is expressed more in MDA-MB-468 cells than MDA-MB-231 cells (>3 fold, P < 0.01, n = 6; 10257 ± 212 vs. 3408 ± 136 pg/mg) or MCF-7 cells (>30 fold, P < 0.01, n = 6; 10257 ± 212 vs. 336 ± 15 pg/mg). Clearly, VEGF expression in TNBC cells is much higher than estrogen receptor positive cells (MCF-7). These results may suggest that VEGF in breast cancer could be biological marker for breast cancer prognosis and progression.

### Sunitinib suppresses the proliferation of cultured MDA-MB-468 or MDA-MB-231 cells

We used a 3H-thymidine incorporation assay to determine the effects of sunitinib on the proliferation of cultured MDA-MB-468 cells. Figure 3B shows that treating MDA-MB-468 cells with sunitinib causes a dose-related decrease in 3H-thymidine incorporation, decreasing by 24% at 1 μmol/L, by 41% at 5 μmol/L, and 59% at 10 μmol/L, compared to the control group (n = 6; P < 0.01), respectively. Also, sunitinib-treatment caused a dose-related inhibition on proliferation in cultured MDA-MB-231 cells, by 23% at 1 μmol/L, by 40% at 5 μmol/L, and 55% at 10 μmol/L, compared to the control group (n = 6; P < 0.01), respectively (Figure 2C). The findings suggest that sunitinib can inhibit proliferation by directly targeting the basal-like or claudin-low TNBC cells.

### Sunitinib directly inhibits migration and increases apoptosis of cultured MDA-MB-468 cells

**Figure 4:**
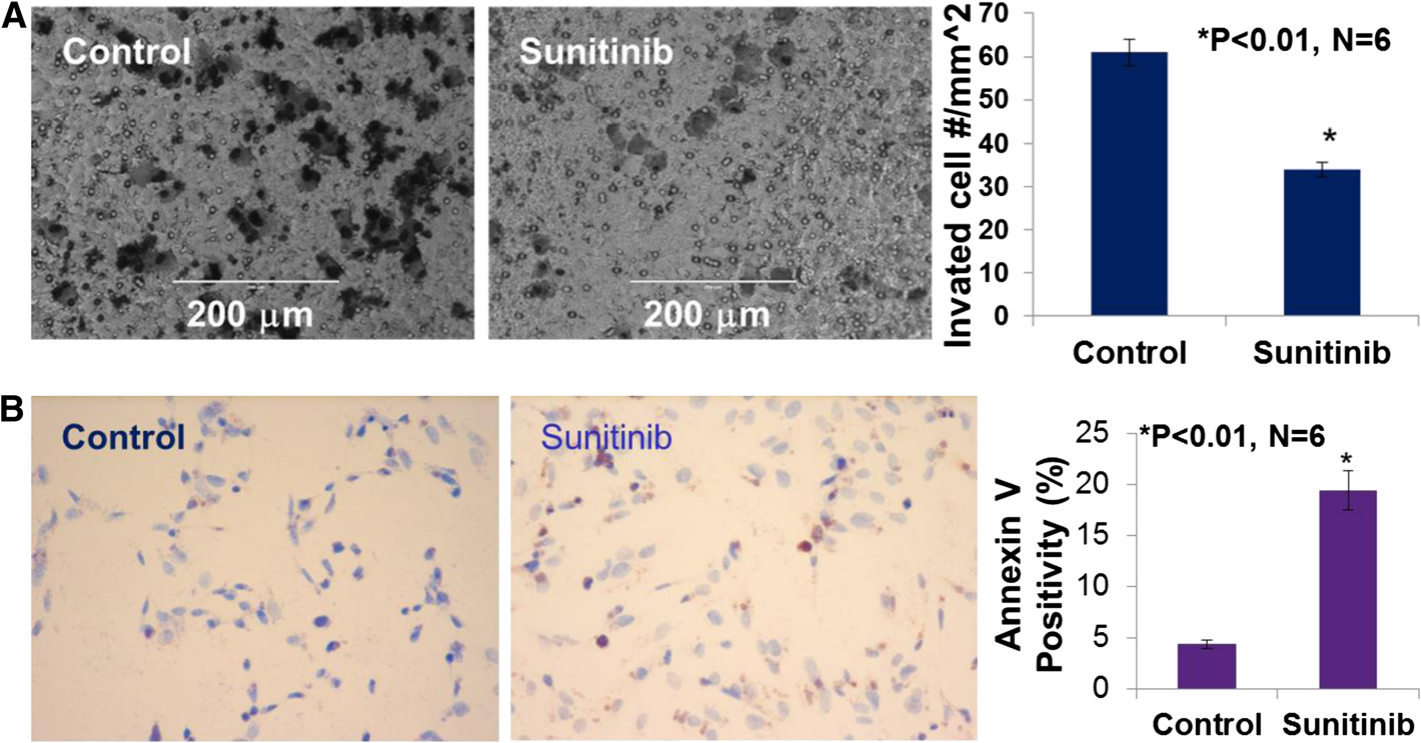
**Sunitinib at 1 μmol/L significantly inhibited the invasion of MDA-MB-468 cells invasion or migration in BD BioCoat Matrigel Invasion Chamber, compared to the control group (34 ± 4 vs. 61 ± 8 cell number/mm**^**2**^**; P < 0.01; n = 6).** The images showed the migrated MDA-MB-468 cells **(A) (B)** indicated that sunitinib at 5 μmol/L significantly increased apoptosis of cultured MDA-MB-468 cells. The images were TUNEL staining of sunitinib-treated or the control MDA-MB-468 cells. Anuexin V-positive cells were observed in sunitinib-treated group, compared to the control group (19.4% vs. 4.4% of Anuexin V-positive cells; n = 6; P < 0.01), respectively.

We examined the inhibitory effect of sunitinib on MDA-MB-468 cell migration using BD BioCoat Matrigel Invasion Chamber. Figure 4A demonstrated that sunitinib at 1 μmol/L significantly inhibited the invasion of MDA-MB-468 cells by 45% compared to the control (n = 6; P < 0.01). In the another experiment, as shown in Figure 4B, we demonstrated that sunitinib at 5 μmol/L significantly increased apoptosis of cultured MDA-MB-468 cells, in which increased TUNEL staining (Figure 3B images) and Anuexin V-positive cells were observed in sunitinib-treated group, compared to the control group (19.4% vs. 4.4% of Anuexin V-positive cells; n = 6; P < 0.01), respectively. These results suggest that sunitinib can directly target the basal-like TNBC cells to inhibit migration and increase apoptosis.

### Sunitinib-treatment *in vivo* significantly increases the percentage of breast cancer stem cells in the basal-like or claudin-low TNBC

**Figure 5:**
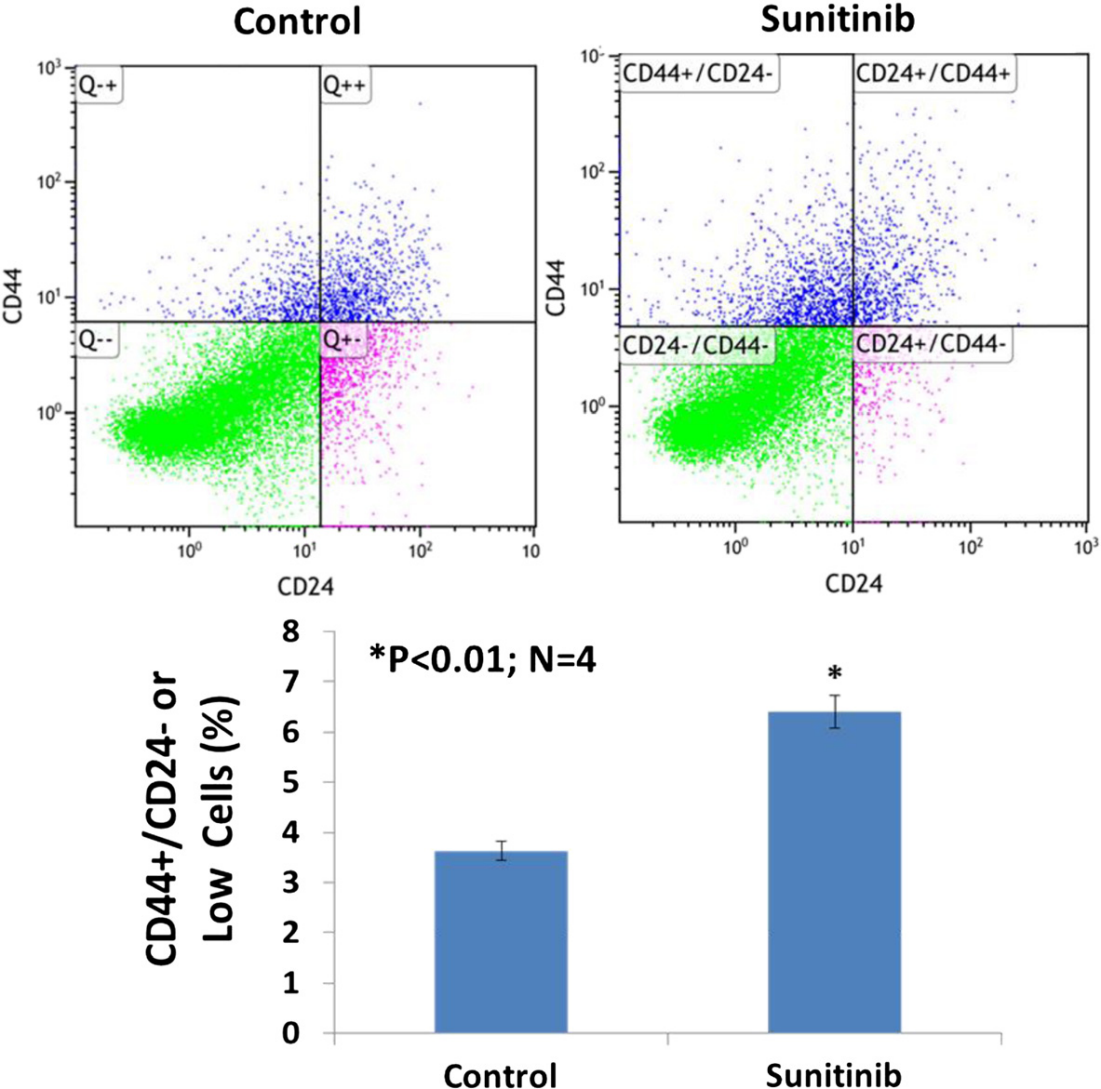
**Flow cytometry analysis of the tumor cells stained with anti-human CD44-PE/CD24-FITC indicated that sunitinib treatment *in vivo *** **significantly increased the percentage of breast cancer stem cells (CD44+/CD24- or low) in basal like breast cancer (MDA-MB-468) in athymic nude-foxn1 mice (3.6 ± 0.3% vs. 6.4 ± 0.5%; n = 4; P < 0.01).**

To determine whether sunitinib stimulates an increase in breast cancer stem cells *in vivo*, the tumor cells in a single cell suspension were isolated from the each tumor in the sunitinib-treated or the control MDA-MB-468/xenografts 4 weeks after the treatment. Flow cytometry analysis of the tumor cells stained with anti-human CD44-PE/CD24-FITC indicated that sunitinib treatment *in vivo* significantly increased the percentage of breast cancer stem cells (CD44+/CD24- or low) in basal like breast cancer (MDA-MB-468) in athymic nude-foxn1 mice (3.6 ± 0.3% vs. 6.4 ± 0.5%; n = 4; P < 0.01) as shown in Figure 5. Treatment with sunitinib for 28 days initiated after MDA-MB-231 tumors reached around 500 mm^3^ significantly increased the percentage of Aldefluor-positive tumor cells (breast CSCs), by 2.3-fold compared to the control group (3.4 ± 0.8% vs. 1.5 ± 0.7%; P < 0.01; N = 4). The results of sunitinib on MDA-MB-231xenografts were consistent with the previous report by Conley SJ et al. [17]. These findings suggest that sunitinib increases breast cancer stem cells in TNBC *in vivo*.

### Sunitinib increases the expression of Notch-1 protein in cultured MDA-MB-468 or MDA-MB-231 cells

**Figure 6:**
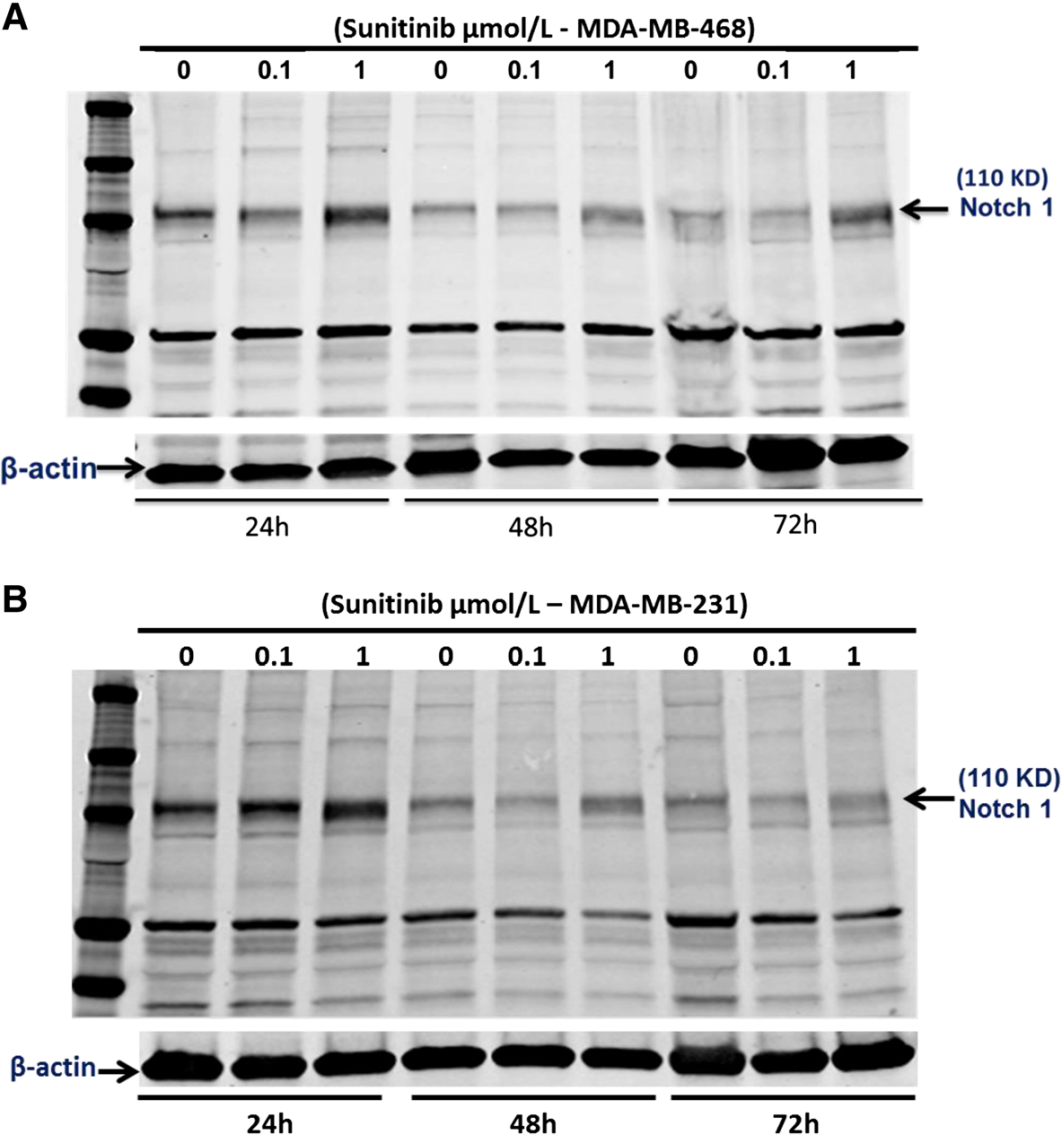
**Western blot analysis indicated that sunitinib at 1 μmol/L significantly increased the expression of Notch-1 at 24, 48, and 72 hours of the treatment in cultured MDA-MB-468 cells (A) and MDA-MB-231 cells (B), respectively.** In cultured MDA-MB-468 cells, compared to the control group, respectively (n = 4; P < 0.01), in which the densitometry ratio of Notch1/β-actin in sunitinib-group was significantly (P < 0.01) increased by 2.0-fold, 2.5-fold, and 5.7-fold at 24, 48, and 72 hours than the control group, respectively. But, sunitinib at 0.1 μmol/L had no effect on the expression of Notch-1. The similar results were also observed in cultured MDA-MB-231 cells.

Notch signaling has been proposed to maintain the stemness of breast cancer stem cells [25,26]. Elevated Notch-1 in human breast cancer is associated with poor clinical outcomes [33]. To determine the possible mechanisms of sunitinib-induced the stemness of breast cancer stem cells, we used Western blot for examining whether sunitinib increases the expression of Notch1 in cultured MDA-MB-468 cells. Cultured MDA-MB-468 cells were treated with sunitinib (0.1 and 1 μmol/L) or the vehicle for 24, 48, and 72 hours. Sunitinib at 0.1 μmol/L did not significantly increase the expression of Notch-1 at 24, 48, and 72 hours of the treatment compared to the control group, respectively (n = 4; P > 0.05) as shown in Figure 6. However, in Figure 6A, sunitinib at 1 μmol/L significantly increased the expression of Notch-1 at 24, 48, and 72 hours of the treatment compared to the control group, respectively (n = 4; P < 0.01), in which the densitometry ratio of Notch1/β-actin in sunitinib-group was increased by 2.0-fold, 2.5-fold, and 5.7-fold at 24, 48, and 72 hours of the treatment compared to the control group, respectively. The similar results of sunitinib increasing Notch 1expression were also observed in cultured MDA-MB-231 cells (Figure 6B). Interestingly, sunitinib at 1 μmol/L significantly increases the expression of Notch-1 in cultured MDA-MB-468 and MDA-MB-231 cells, which may be associated with increasing breast CSCs.

## Discussion

The major new findings from this study include: 1) VEGF is highly expressed in basal-like breast cancer cells (MDA-MB-468); 2) sunitinib significantly inhibits the proliferation, invasion, and apoptosis resistance in cultured basal like breast cancer cells; 3) sunitinib significantly reduces tumor volume of basal like breast cancer in nude mice in association with the inhibition of tumor angiogeneisis; 4) sunitinib increases breast cancer stem cells *in vivo*; and 5) sunitinib significantly increases the expression of Notch1 in cultured MDA-MB-468 cells. Though sunitinib inhibits the progression of basal-like breast cancer by directly targeting both tumor cells and vasculature the possibility should be considered that it may increase breast cancer stem cells. In addition, the present studies confirm the previous report that sunitinib inhibited tumor angiogenesis and growth in claudin-low TNBC (MDA-MB-231) xenografts, but increased percentage of breast cancer stem cells [17].

TNBCs are comprised of both the basal and claudin-low molecular subtypes. The majority of TNBCs (approximately 80%) are the basal-like breast cancers [4]. Also, 12% of the TNBC patients (16/132) have claudin-low (normal-like) subtype [34]. The basal-like breast cancer subtype is best identified by DNA microarray expression profiling, but this methodology is not readily available in clinical practice [35]. In a phase II study of patients with heavily pretreated metastatic breast cancer, 15% of patients (three of 20) with TNBC achieved partial responses following treatment with single-agent sunitinib [18]. It is not clinically know whether sunitinib is effective in the basal or claudin-low molecular subtypes. Previous studies [17,36,37] showed that sunitinb alone significantly inhibited tumor growth in the claudin-low TNBC (MDA-MB-231) xenografts. The present study demonstrates that the treatment with single-agent sunitinib is very effective in the inhibition of the basal-like breast cancer progression by directly targeting both of tumor cells and tumor vasculature using MDA-MB-468 xenografts and cultured cells. These findings combined with the data of sunitinib on MDA-MB-231 xenografts suggest that sunitinib is effective in the treatment of TNBCs including the basal and claudin-low molecular subtypes.

VEGF has been shown to be highly expressed in breast tumors at levels that are 7-fold higher than normal adjacent tissue [38]. The median level of intratumoral VEGF expression in the TNBC population is significantly higher than the non-TNBC population (8.2 vs. 2.7 pg/μg DNA; P < 0.01), in which TNBC patients have a significantly worse relapse free survival, earlier distant recurrences, and a shorter time between relapse and death, compared with the non-TNBC group [39]. Although the median values for VEGF between the TNBC and the non-TNBC are significantly different, the ranges for both groups are large [39], implying heterogeneity within the groups. In the present study, we have found that the VEGF values are wildly different between cultured MCF-7 cells (336 ± 15 pg/mg), MDA-MB-231 cells (3408 ± 212 pg/mg), and MDA-MB-468 cells (10257 ± 136 pg/mg). Even within different TNBC cell lines, the VEGF values in basal-like (MDA-MB-468) cells are 3-fold higher than claudin-low (MDA-MB-231) cells. The potential role of intratumoral VEGF expression levels in clinical practice remains unclear; however, VEGF has emerged as a potential therapeutic target in a number of solid malignancies, including breast cancer. High levels of VEGF expression have been associated with poor clinical outcome in many solid tumors [39,40]. We assume that sunitinib could be more sensitive to the breast tumors with highly expressed VEGF than the breast tumors with low expressed VEGF. In the future, we will compare the different responses to sunitinib in treating breast cancer using MCF-7, MDA-MB-231, and MDA-MB-468 xenografts.

The *in vivo and in vitro* findings from this study suggest that sunitinib targets the basal-like breast cancer tumor vasculature as well as the tumor epithelial cells directly. The signal-transduction pathways involving vascular endothelial growth factor receptor (VEGFR), platelet-derived growth factor receptor (PDGFR), stem-cell factor receptor (KIT), and colony stimulating factor-1 receptor (CSF-1R) have been implicated in breast cancer pathogenesis [5-10]. VEGFR and KIT have shown to be associated with TNBCs [10-13]. Sunitinib is an inhibitor of receptor tyrosine kinases that include VEGFR, PDGFR, KIT, and CSF1R [6,11,14]. Though it is possible to antagonize VEGFR by sunitinib, targeting of other receptors may contribute to the activity of the agent. Preclinical studies across multiple cell lines have demonstrated IC50 values in the nanomolar range for c-kit, flt3 and RET [41]. Thus, VEGFR antagonism alone may not fully explain the antitumor effect of sunitinib.

In the present study, oral sunitinib at 80 mg/kg/2 days for 4 weeks very significantly inhibits tumor growth in the basal-like TNBC (MDA-MB-468) xenografts, but it significantly increases the percentage of breast cancer stem cells (CSC) in the tumors. The connection between reduced tumor angiogenesis/tumor growth, and increased CSC by sunitinib is of interest. These findings support the notions: 1) antiangiogenic therapies in breast cancer show some therapeutic potential with increased disease-free survival; and 2) these initial promising results are short lived and followed by tumor progression, regrowth, and more aggressive and invasive tumors [42]. CSCs are thought to play a role in recurrence and metastasis of TNBC [25]. CSCs are predicted to be the cell origin of the tumor and responsible for tumor progression, relapse and metastasis due to their self-renewal capacity and limitless proliferative potential, as well as invasion and migration capacity [43]. Although CSCs comprise a small amount of the cells within a tumor, they can be resistant to radiotherapy and chemo-therapeutic agents, probably because of their quiescence. Therefore, the development of successful cancer therapy requires targeting the CSCs. We would like to develop the TNBC therapeutic regimen with sunitinib plus anti-CSC agent.

Increased CSC by sunitinib is possibly due to increased intratumoral hypoxia that has been linked to the stimulation of cancer stem cells (CSC) [23,24]. Hypoxia-inducible factor-1 (HIF-1) has been implicated in the maintenance of cancer stem cells, although the specific HIF target genes involved in this process have not been identified [17,44]. Our data on increased CSC by sunitinib in the basal-like TNBC (MDA-MB-468) xenografts support the previous findings that antiangiogenic agents increase breast cancer stem cells via the generation of tumor hypoxia [17].

In studies of stem and/or progenitor cells isolated from the mammary gland, Notch pathway has been implicated in self-renewal of stem cells, maintaining stem cell potential and inhibition of differentiation [25]. The experiments support that the Notch pathway is critical in controlling the fate of CSC in breast cancer [25,26]. Higher expression of Notch-1 and its ligand Jagged-1 is associated with poor prognosis in breast cancer [33]. Moreover, studies have suggested that Notch-1 could play a key role in the regulation of EMT and CSC phenotype during the development and progression of tumors [45,46]. The present study shows a new finding that sunitinib significantly increases the expression of Notch-1 in culture MDA-MB-468 cells as well as MDA-MB-231 cells even under the normoxia condition, which is consistent with increased CSC by sunitinib in the basal-like TNBC (MDA-MB-468) or the claudin-low TNBC xenografts. These results support the hypothesis that the anti-angiogenic therapy may actually activate Notch and preserve CSC [27]. The further studies are necessary to investigate the mechanisms of sunitinib-induced up-regulation of Notch-1. However, sunitinib plus γ-secretase inhibitor (Notch inhibition) in breast cancer therapy could be the innovative therapeutic strategies that simultaneously target angiogenesis and CSC.

## Conclusion

In conclusion, our results indicate that oral administration of sunitinib, an inhibitor of receptor tyrosine kinases that include VEGFR, PDGFR, KIT, and CSF1R, significantly inhibits tumor growth and tumor angiogenesis in basal-like TNBC (MDA-MB-468) or claudin-low TNBC (MDA-MB-231) xenografts that highly express VEGF. Sunitinib also directly targets the tumor epithelial cells inhibiting proliferation and migration, and increasing apoptosis. Increased breast cancer stem cells by sunitinib *in vivo* are possibly due to increased intratumoral hypoxia and the up-regulation of Notch pathway. These findings suggest that sunitinib alone is effective but not good enough for treading TNBC. On the other hand, in combination with the results of sunitinib-increased CSCs and Notch-1 expression, this work provides the framework for development of innovative therapeutic strategies in TNBC therapy by using sunitinib plus γ-secretase inhibitor to simultaneously target angiogenesis and CSC. Further studies are necessary to investigate how combination therapy may improve TNBC treatment.

## Competing interests

The authors declare that they have no competing interests.

## Authors’ contributions

JG prepared the manuscript and all figures. EC did the experiments of MDA-MB-468 xenografts and cell cultures. KM measured capillary density using CD31 immunohistochemistry. JG did the apoptosis assays and migration assays. FC and SC did the experiments of MDA-MB-231 xenografts and cell cultures. GM, SV, and LM reviewed and edited the final manuscript. All authors read and approved the final manuscript.
